# Development and design of a culturally tailored intervention to address COVID-19 disparities among Oregon's Latinx communities: A community case study

**DOI:** 10.3389/fpubh.2022.962862

**Published:** 2022-09-23

**Authors:** Elizabeth L. Budd, Ellen Hawley McWhirter, Stephanie De Anda, Anne Marie Mauricio, Maryanne V. Mueller, Camille C. Cioffi, Ashley Nash, Kelsey Van Brocklin, Kristin Yarris, Arriell Jackson, Heather Terral, Jorge I. Ramírez García, Juntos Podemos Community and Scientific Advisory Board, William A. Cresko, David S. DeGarmo, Leslie D. Leve

**Affiliations:** ^1^Department of Counseling Psychology and Human Services, University of Oregon, Eugene, OR, United States; ^2^Prevention Science Institute, University of Oregon, Eugene, OR, United States; ^3^Department of Special Education and Clinical Sciences, University of Oregon, Eugene, OR, United States; ^4^Department of Global Studies, Center for Global Health, University of Oregon, Eugene, OR, United States; ^5^Institute of Ecology and Evolution, University of Oregon, Eugene, OR, United States; ^6^Presidential Initiative in Data Science, University of Oregon, Eugene, OR, United States

**Keywords:** COVID-19 testing, Hispanic Americans, health promotion, minority health, health status disparities, Latinx

## Abstract

**Background:**

Latinx communities are disproportionately affected by COVID-19 compared with non-Latinx White communities in Oregon and much of the United States. The COVID-19 pandemic presents a critical and urgent need to reach Latinx communities with innovative, culturally tailored outreach and health promotion interventions to reduce viral transmission and address disparities. The aims of this case study are to (1) outline the collaborative development of a culturally and trauma-informed COVID-19 preventive intervention for Latinx communities; (2) describe essential intervention elements; and (3) summarize strengths and lessons learned for future applications.

**Methods:**

Between June 2020 and January 2021, a multidisciplinary team of researchers and Latinx-serving partners engaged in the following intervention development activities: a scientific literature review, a survey of 67 Latinx residents attending public testing events, interviews with 13 leaders of community-based organizations serving Latinx residents, and bi-weekly consultations with the project's Public Health and Community Services Team and a regional Community and Scientific Advisory Board. After launching the intervention in the field in February 2021, bi-weekly meetings with interventionists continuously informed minor iterative refinements through present day.

**Results:**

The resulting intervention, *Promotores de Salud*, includes outreach and brief health education. Bilingual, trauma-informed trainings and materials reflect the lived experiences, cultural values, needs, and concerns of Latinx communities. Interventionists (21 Promotores) were Latinx residents from nine Oregon counties where the intervention was delivered.

**Conclusions:**

Sharing development and intervention details with public health researchers and practitioners facilitates intervention uptake and replication to optimize the public health effect in Oregon's Latinx communities and beyond.

## Introduction

U.S. born and immigrant Hispanic/Latino/a/x [henceforth referred to as Latinx; (1)] persons have been disproportionately affected by the COVID-19 pandemic (2, 3). In the early months, Latinx and Spanish-speaking individuals were both less likely to get tested, and more likely to test positive for SARS-CoV-2 — the virus that causes COVID-19 (4, 5). Latinx residents constitute 13.9% of the population in Oregon (6), but in Mid-May of 2020 accounted for 31.7% of the state's COVID-19 cases (7). By July of 2020, weekly cases for Oregon's Latinx residents were 174.7 per 100,000, and only 28.1 per 100,000 for non-Latinx Whites (8). The mortality rate was also higher for Latinx residents even though Oregon's Latinx population is much younger than the non-Latinx White population (e.g., median age of 24 vs. 41 years, respectively) (9, 10). Testing for SARS-CoV-2 is critical to prevention and control (11). This case study describes the development and essential elements of an innovative culturally and trauma-informed intervention designed to increase SARS-CoV-2 testing, and decrease transmission, among Latinx communities in Oregon.

## Background and rationale

### The need for tailored interventions for Latinx community members

The Latinx population of Oregon has grown by 31% since 2010 and is the largest ethnically minoritized group in the state (6). The majority (85%) are of Mexican origin, and 70% speak a language other than English at home (10), predominantly Spanish, but also including 0.6% who speak Mam, Mixtec, Tzotzil, or another indigenous language of Mexico or Central America (12). Oregon's Latinx residents are much more likely to live in poverty, lack health insurance, and experience food insecurity than non-Latinx Whites (10, 13). Oregon's undocumented immigrants, 77% of whom are Latinx (14), are often employed in what the Department of Homeland Security deemed “critical infrastructure” roles — such as agriculture and food processing — and these workers have been disproportionately affected by job loss, contagion, and death during the pandemic (15) but are not eligible for federal pandemic stimulus benefits (16, 17). Lack of health insurance, pre-existing health conditions, employment as essential workers, lack of access to protective gear, and language barriers increase vulnerability to COVID-19 (18). Efforts to mitigate SARS-CoV-2 spread among Latinx communities must account for these factors and provide access to testing (2, 4, 5, 19, 20).

Ongoing inequities in healthcare and education (2), chronic stress associated with anti-immigration policies (21), and trauma from the disproportionate effects of COVID-19 including inadequate workplace protections from the virus, sudden job loss, hospitalization, and death combine with sociopolitical histories of oppression to decrease trust in government authorities and programs and necessitate trauma-informed approaches to engage Latinx communities in SARS-CoV-2 testing and prevention efforts (2, 17, 21). Trauma-informed approaches to behavioral health services recognize the prevalence of trauma, how it is manifested, and its multifaceted effects on individuals and communities, emphasize physical, psychological, and emotional safety for both users and providers of the services, promote and support user agency, and utilize practices and procedures that minimize the likelihood of re-traumatization (22). We developed and delivered our innovative health promotion intervention using a trauma-informed approach, as recommended but little researched with Latinx communities (23).

### Case study purpose

Disparities in SARS-CoV-2 testing and contagion showed that Oregon Latinx communities were not being served by standard outreach and health promotion practices. Our team aimed to develop an innovative culturally tailored and trauma-informed intervention to increase SARS-CoV-2 testing and engagement in preventive behaviors among Oregon Latinx communities. A community-based participatory research (CBPR) approach was followed as much as possible by soliciting and incorporating experiences, ideas, and feedback from community stakeholders, who were members of the communities we aimed to serve, at every step of the intervention development and refinement process to promote knowledge transfer, ownership of the intervention's success, and sustainability (24–26). The purpose of this case study is to describe intervention development activities and the resulting *Promotores de Salud* intervention to facilitate replication and optimize potential public health effects. Integrated throughout are the items from the GUIDED (GUIDance for the rEporting of intervention Development) checklist by Duncan and colleagues (27) and Smith, Levkoff and Ory's (28) list of key components of a community case study.

## Methods

This project, entitled Oregon Saludable: Juntos Podemos, was part of a national effort to increase SARS-CoV-2 testing among underserved populations funded through the National Institutes of Health Rapid Acceleration of Diagnostics for Underserved Populations (RADx-UP) initiative. The request for RADx-UP proposals was made in June 2020 and notice of award in September 2020. Community leaders who later became members of the Community and Scientific Advisory Board (CSAB) or Public Health and Community Services Team provided input on the study design and approach. A multidisciplinary team of researchers from public health, language and communication sciences, counseling psychology, prevention science, and sociocultural anthropology at the University of Oregon led the intervention development and implementation in close collaboration with a multi-sector team that spanned the state, including researchers, community-based organizations (CBOs), local and state health departments, and a CSAB. The design of the larger study included random assignment of testing sites within counties to intervention or outreach-as-usual conditions. Primary intervention development activities were informed by CDC recommended community assessment methods for promoting health equity, including reviewing existing data and evidence and conducting surveys and interviews (29). These primary intervention development activities occurred between June 2020 and January 2021 (Table 1). The intervention was launched in the field in February 2021, and refinement continues with input from our stakeholder groups. All study activities that involved human subjects were approved by the University of Oregon Institutional Review Board (IRB).

**Table 1:** Timeline of Promotores de Salud development and implementation.

**Year**	**2020**				**2021**								
**Months**	**May/June**	**July/Aug**	**Sept/Oct**	**Nov/Dec**	**Jan/Feb**	**March/April**	**May/June**	**July/Aug**	**Sept/Oct**	**Nov/Dec**	**May/June**	**July/Aug**	**Sept/Oct**
Literature review													
Survey of Latinx community members													
Key stakeholder interviews													
Community and Scientific Advisory Board consultations													
Public health and community services team consultations													
Intervention implementation													
Meetings with Promotores													

### Activity 1: Literature review

In Summer 2020, the first author and two research assistants conducted a review of the scientific literature between 2005–2020 on culturally tailored public health and medical interventions for Latinx patients or communities in the United States. The following MeSH terms were included in PubMed and Google Scholar searches: Hispanic or Latino, United States, prevention and control, cultural competency. Reports on best public health or medical practices among Hispanic or Latino/a/x individuals by organizations such as the CDC, U.S. Department of Health and Human Services Office of Minority Health, and American Psychiatric Association were also included in the review. Research assistants created an annotated bibliography focusing on evidence-supported intervention and outreach practices related to caring for and reaching Latinx community members and noted theoretical frameworks that informed this literature.

### Activity 2: Survey of Latinx community members

Between July and October 2020, a researcher on the CSAB and a research assistant visited all nine SARS-CoV-2 testing events, organized by the Lane County (Oregon) Public Health Department, that targeted Latinx and Spanish speaking community members through outreach in Spanish and through Latinx-serving CBOs. Using convenience sampling, community members were approached while they waited in line for testing. If a family was in line together, the researcher asked if one of the adults would be interested in hearing about the survey. The survey purpose and procedure were explained in the preferred language (Spanish or English) expressed by the community member. If interest was expressed, community members' eligibility was confirmed (≥ 18 years and identified as Hispanic or Latino/a/x) and they were consented and surveyed after they completed SARS-CoV-2 testing. Approximately five eligible adults who were approached about the study declined to participate. No incentives were provided for participation. Sixty-seven Latinx community members completed the 3–5-min survey using the Epicollect5 (https://five.epicollect.net) app. The final sample size was the maximum number of eligible adults the investigator was able to invite to the participate, given the time it took to conduct each informed consent and survey, minus the individuals who declined across visited testing events.

The 11-question survey was informed by the Health Belief Model (e.g., perceived benefits, barriers, and threat), feedback from local health department practitioners, and questions/concerns expressed by community members through the local COVID-19 public hotline. The survey assessed: how participants heard about the testing events; from what sources they obtain COVID-19 information; reasons for seeking testing; perceptions of why others in their community may be reluctant to seek testing; ways the pandemic had affected themselves and their families; the industry in which they primarily work; and whether participants had health insurance, were employed, had lost a job due to the pandemic, were exposed to COVID-19 risks at their job, and were aware of state financial resources available to those who had lost a job or had been adversely affected by the pandemic. The researcher chose not to collect additional socio-demographic information in the survey in order to receive the fastest possible IRB review and approval. This trade-off allowed for the data collection at these limited-time events but prohibited any assessment of who is or is not represented in the sample beyond that participants were all adults, Latinx, and opted to participate in Spanish.

### Activity 3: Key stakeholder interviews

Between July and September 2020, key community stakeholders (defined as leaders of CBOs that predominantly serve Latinx community members in Lane County) were purposely selected and invited to participate in interviews. The purpose of these interviews was to inform COVID-19 testing event protocols and related communications to maximize acceptability, feasibility, and reach among Latinx community members. Participating stakeholders underwent informed consent and completed a 45–60-min, one-on-one interview *via* Zoom. Informed by Guest, Namey, and Chen's (16) recommendations, the study team determined that thematic saturation was reached within the 13 completed interviews. Three research assistants, trained and supervised by the first-and sixth author, carried out all informed consent processes, interviews, and analyses in English.

The semi-structured interview guide included 44 questions informed by the Elaboration Likelihood Model of Persuasion (30). First, demographic information was assessed on the stakeholders and community members they serve. Then, stakeholders' feedback on three primary domains was gathered, including (1) testing event messaging and communication channels for promoting testing events; (2) on-site testing event logistics, protocols, and staff; and (3) methods for sharing of test results. Stakeholders responded to each interview question, first reflecting their own preferences, and then reflecting those of the community members they serve. Importantly, the latter questions aimed to learn from the stakeholders' expertise in serving Latinx community members, rather than serving as a proxy for Latinx community members' voices.

The interviews were audio-recorded and transcribed using Zoom, transcriptions were edited by research assistants, and analyzed using a conventional content analytic approach, starting with the interview guide questions as an a priori framework (31). After achieving consensus on the identified themes, a final review of transcripts was conducted to identify any potential additional themes that were distinct from or counter to existing themes; none were identified.

### Activity 4: Community and Scientific Advisory Board consultations

To provide guidance on the community, cultural, and linguistic responsiveness of the research study, a CSAB was established in September 2020. The CSAB met monthly *via* Zoom, from September 2020 to the present, to review and provide feedback on study operations and materials. CSAB consisted of eight individuals with expertise in COVID-19 public health response at the state and county levels, inclusive of members of Oregon's Health Equity Committee, research investigators who specialize in Latinx populations, and members of CBOs and behavioral health organizations with experience working with immigrant and Latinx families. By design, CSAB members had high levels of Spanish proficiency, included young and middle-aged adults, had work and/or lived experiences with Latinx communities across different regions of Oregon, and had heritages in various Latin American countries. At least one investigator and staff person from the research team also attended the CSAB meetings. Examples of agenda items included review of intervention materials and Promotores' training materials.

### Activity 5: Public health and community services team consultations

October 2020 through August 2021, 2–3 members of the research team met weekly with a group of 5–8 Lane County Latinx-serving social services providers, including individuals from a local CBO and from the local health department's Latinx Outreach Team. These professionals were Spanish-English bilingual and identified as Latinx. Meetings took place over Zoom and followed an agenda co-established at the beginning of each meeting. Initial meetings focused on the purpose of the intervention, constraints and resources associated with the project, and the initial vision for the intervention. As the intervention evolved, the associated materials, trainings, content, and scripts were presented iteratively to this team and feedback was incorporated.

### Activity 6: Meetings with Promotores

From February 2021 through present, approximately two interventionists (Promotores) in each of nine counties were hired to deliver the intervention. The research team contracted with partner CBOs, and CBOs hired the Promotores. Although the research team offered hiring guidelines (connected to local community, bicultural, Spanish and English-speaking), CBOs hired Promotores independent of the research team. Because these CBOs were well-established and predominately served local Latinx community members, they had experience hiring employees responsive to community members' needs. All Promotores identified as Hispanic or Latino/a/x and were connected to their counties' Latinx communities, largely by residence and/or employment. Approximately 44% of the Promotores had a high school diploma or GED, 19% had a college degree, 13% had some college, 6% did not report; annual income ranged from $4,500–$60,000 (*M* = $27,375, *SD* = $14,160); 50% were born in the United States; 20% had a parent born in the United States; 25% reported Spanish as their primary language, 31%, English, and 44% both English and Spanish equivalently. Promotores participated in bi-weekly meetings *via* Zoom, facilitated by several investigators on the research team, in order to continuously refine the intervention for optimal implementation and effectiveness. During these meetings, investigators solicited interventionists' feedback on barriers and facilitators to intervention delivery, and possible adaptations, clarifications, or additional materials that would improve intervention delivery and/or better serve the needs of the Latinx residents in their counties. These meetings also offered a space to collaboratively review intervention materials, practice and receive feedback on Promotores' delivery of the intervention, and receive support related to implementation challenges. Promotores also had the opportunity to reflect on ways in which their own stress, traumas, and lived experiences more generally might impact them in their role when engaging with community members. Additional check-ins provided space for Promotores to share in a smaller group setting and address more county and Promotor-specific issues. In addition, research team members reached out to each partner CBO and affiliated Promotores at least bi-weekly via email or Zoom to answer questions, help problem solve, and exchange ideas.

## Results

Table 2 summarizes the key findings generated by each activity reported in the Methods section and how the information informed the *Promotores de Salud* intervention. The first three activities (the literature review, survey of Latinx community members, and interviews with key stakeholders), were conducted prior to initiating development of the intervention. The latter three activities (CSAB consultations, Public Health and Community Services Team consultations, and Promotores meetings) reflect ongoing iterative processes through which feedback was sought and incorporated over time as the intervention was implemented in the field. While the survey and interview data were collected among residents of Lane County, continuous input from Promotores and CSAB members ensured that intervention development was sensitive to contextual differences across counties. *Promotores de Salud* was associated with three and a half more Latinx community members engaged in testing compared with outreach-as-usual sites, representing a medium-to-large effect size (0.70). Evaluation results and how testing sites were selected to optimize convenience and familiarity to Latinx communities are reported elsewhere (43, 44). Details of the implementation approach, guiding frameworks, and findings will be outlined in forthcoming articles.

**Table 2:** Summary of results from intervention development activities.

**Development activity**	**Result highlights with examples of how findings were translated to the intervention**
1. Literature review	• Employ Latinx community health workers, “Promotoras” (32, 33).
	• Recruit, mentor, and promote bicultural and bilingual Latinx staff at all levels, use community health workers to support engagement and outreach, and engage experienced translators (32).
	• Training of Promotores should promote shared decision making, open communication, and trust (34).
	• Engage principles of trauma-informed care in the intervention and the training (21, 22, 35).
	• Target health communication with bilingual messaging, multiple outlets (e.g., radio and television), and collaborate with trusted community organizations (36, 37).
	• Consider acculturation, community, family connections, immigration status/history, and education (38).
	• Consider testing at schools and student health centers (39).
	• Use health belief model to inform intervention (40, 41).
	• Use Motivational Interviewing to address hesitancy and increase motivation about testing and practicing prevention behaviors (42).
	• Health communication messaging recommendations (37): bilingual messaging, dissemination *via* radio and television, attend to cultural meanings and variations, consider generational and immigration status, acculturation, and assimilation, attend to cultural values such as marianismo and machismo in communication, collaborate with trusted organizations that serve Latinx communities, understand the importance of traditional healing systems (for cultural and financial reasons) and the cultural and linguistic heterogeneity of the communities, and address barriers to healthcare seeking.
	**Translation to intervention**
	• Promotores were hired and trained as interventionists. They were Latinx community members residing in or familiar with the communities they were serving.
	• Trusted, Latinx-serving community-based organizations were invited as collaborators and informed test site decisions (accessible and acceptable locations).
	• Outreach, education materials, and protocols guiding Promotores-participant interactions were informed by Latinx cultural and social values and norms and embedded trauma-informed care and Motivational Interviewing principles, as well as Health Belief Model constructs (e.g., addressing benefits and barriers, and supporting self-efficacy).
	• Materials were in Spanish and English and reviewed by multiple parties for clarity and linguistic variation and nuance; Mam speakers were onsite to translate as needed.
	• Materials and Promotor training reflected cultural values.
	• Intervention included resource navigation support to address economic and other barriers and support self-efficacy.
	• Outreach leveraged community networks and targeted settings to which Latinx community members were already connected (churches, schools, local businesses).
2. Survey of Latinx community members (*N* = 67)	• Most respondents (64%) obtain information about COVID-19 through social media, followed by local Spanish-language radio (58%).
	• Respondents received information about free testing events primarily through trusted local community organizations (40% of respondents), followed by word of mouth (30%) and Facebook (19%).
	• The most frequently mentioned motivations for getting tested included preventing the spread (63% of respondents), protecting the family (31%), and for personal knowledge (31%).
	• Perceived barriers to testing in the community included fear of the test itself (68%) and structural barriers such as lack of money, access, or insurance (37% of respondents).
	**Translation to intervention**
	• Partnerships between county health departments and trusted community-based organizations were developed with consistent meetings to elicit feedback and guidance.
	• Outreach strategies included dissemination of information about testing events *via* trusted community organizations and leaders (e.g., churches) and directly, using social media, Spanish language radio, and flyers.
	• Outreach and on-site interactions emphasized how testing is a way to care for families, self, and others, aligning with cultural values.
	• Outreach provided visual and verbal information about the testing process (the less invasive nasal test; later, self-administered tests).
3. Key stakeholder interviews (*N* = 13)	**Recommendations for Communication** • Provide outreach materials and health communications in Spanish or (in certain areas) Mam. Create the materials and communications in Spanish or Mam first, rather than English first and then translating to Spanish or Mam.
	• Have Spanish and Mam-speaking translators at testing sites to serve community members not fluent in English.
	• Share test results with a phone call, and preferably in Spanish.
	**Testing logistics**
	• Persecution of Latinx immigrants has created fear of being detained amongst many Latinx people, even those with legal status. Testing should take place at trusted, known sites to alleviate fear and suspicion.
	• Provide justification for requesting any private information, such as insurance information and home address, as well as how information will be stored.
	• Provide visual barriers in testing sites for privacy concerns.
	**Translation to Intervention**
	• Materials were reviewed for language and clarity by multiple native speakers.
	• Mam-speaking translators were on site as needed; all Promotores were Spanish-fluent.
	• Promotores doing outreach and on site helped alleviate fears and explained the processes and use of information.
	• Safety concerns at any site were addressed immediately.
4. Community and Scientific Advisory Board feedback	**Specific suggestions incorporated:** • Use Promotor (es), rather than Promotora (s); use "Latino” in interactions with the community and “Latinx” only with academic audiences.
	• Use both “metros” (meters) and “pies” (feet) to describe social distance recommendations.
	• Use a single name for the intervention (*Promotores de Salud*) instead of the originally proposed names distinguishing the outreach and intervention elements. (*Promotoras Communitarias* and *Promotoras de Salud*)
	• Minimize text in handouts focused on transmission, for example, use infographics or fotonovelas to make the content more accessible.
	**Outreach suggestions incorporated:**
	• Specific language suggestions for outreach materials to assure they are inviting and engaging to Latinx community members.
	• Provide a phone number for people to ask questions, in order to increase accessibility,
	• Decrease amount of text in outreach materials, change language that requires literacy beyond 6^th^ grade level; add more visuals for community members who are not literate.
	• Add specifics and clarity regarding confidentiality for testing event participants; emphasize that no documentation or insurance is required for testing.
	**Other suggestions incorporated:**
	• Modify language on demographic survey for clarity and specificity (e.g., to capture country of origin); reduce the number of survey items.
5. Public health and community services team feedback	**Intervention/training suggestions incorporated:** • In the brief health education element, include information about vaccinations.
	• Be aware of and ready to counter the myths about COVID-19 and about vaccinations circulating in Latinx communities.
	• Generate possible questions Promotores might be asked by testing participants or during outreach, create a Frequently Asked Questions handout with suggested responses for Promotores.
	• At the close of the brief health education element, include in resource navigation specific information about state resources (state quarantine fund, agricultural worker fund).
	• Increase clarity of forms that the Promotores complete on site.
	• Create and use a transparent process for cancellation of events, to minimize community or Promotor distrust and confusion.
	• Develop a means by which Spanish-speaking monolingual people can ask follow-up questions about their test results (by phone).
	**Outreach suggestions incorporated:**
	• Contact specific radio stations and radio programs, use listservs, and ask specific community organizations to help with outreach.
	• Ask business owners that employ many Latinx people to share fliers with employees (restaurants, sawmill, agriculture).
	• Encourage Promotores to use WhatsApp alerts as a means of outreach if their community-based organizations have an alert system.
	• Change language in fliers from *protejese* and *proteja los dem*á*s* (protect yourself and others) to *cu*í*dese y cuida a los dem*á*s* (take care of yourself and others).
	• Tailor outreach materials (e.g., raffles for game systems to appeal to children and families).
6. Meetings with Promotores	**Intervention suggestions incorporated:**
	• Provide prepared talking points on why testing is recommended, and why testing is recommended after vaccination.
	**Outreach suggestions incorporated:**
	• Broaden outreach activities (e.g., reach out to people at the end of mass on Sundays, ask schools to inform parents of testing events) to reach Latinx community members.
	• Add follow up training related to conducting the outreach and delivering the health education (large and small group discussions about best outreach practices and what to do in various scenarios; create a video role play in Spanish).
	• Create and use door hangers with testing event information.
	• In the outreach video, specify that the COVID-19 test is self- administered and minimally invasive, that all events are free, and add that “everyone is welcome.”

## Essential elements of the intervention

The *Promotores de Salud* intervention had two essential elements: outreach and brief health education. Both elements reflected culturally tailored, evidence- and trauma-informed strategies generated by intervention development activities described above. The intervention elements were delivered by bicultural, Spanish and English-speaking Promotores, similar to community health workers (36). The majority of the 21 Promotores lived in the county in which they served and were intricately connected to the local Latinx community (45), and were hired by partner CBOs that predominantly serve Latinx community members. Employing members of the local Latinx community is a CDC recommended strategy for effective health education (32, 46–48), and Promotores help facilitate the trust and respect necessary for effective study recruitment (48–50). Promotores can be highly effective at recruitment for health-related interventions among all ages of Latinx people (51). Distinct from typical community health worker models, Promotores were only trained on COVID-19 prevention and control, rather than multiple public health topics, and were trained through the research study rather than a standardized training program, such as a state certification (52). Additionally, Promotores tracked data on the reach and fidelity of intervention delivery. Each *Promotores de Salud* element is outlined next, followed by an overview of the training and description of how trauma-informed care and motivational interviewing practices were embedded.

The purposes of the **outreach** element were to build relationships with Latinx community members and with predominately Latinx-serving CBOs and to advertise and promote participation in the SARS-CoV-2 testing events. Promotores shared outreach materials including Spanish and English flyers, door hangers, radio announcements, WhatsApp telephone application messages, and social media messages (e.g., Facebook, Instagram) among Latinx community members directly and through well-established Latinx serving organizations (see Supplementary materials for example). These organizations included grocery stores and Mexican markets, churches, schools, community mental health centers, the regional farmworkers union, and the Women, Infant, and Children program. When relevant, Promotores would ask organizational leaders (e.g., priest, principal) to aid in promoting the testing events among their constituents. Outreach materials included logistical information about when and where testing would occur, that testing was free, and that documentation status was not relevant. Promotores determined the optimal outreach strategies to use in their communities. Promotores verbally reinforced the information on the outreach materials and provided additional details about the testing process (e.g., anterior nares swab vs. nasopharyngeal swab) and what information *would* (e.g., name and date of birth, contact information) and *would not* be requested of them (e.g., proof of citizenship or health insurance). Outreach materials and interactions reflected constructs of the Health Belief Model (40, 41) by emphasizing Latinx community values supportive of testing engagement, acknowledging that Latinx people are hard workers, often essential workers, whose employment necessitates interaction with others, and that testing contributes to collective welfare and provides information to help keep families and the community safe (42).

The purposes of the brief health education element of the intervention were to promote COVID-19 preventive behaviors and to help sustain testing rates of Latinx community members over time. This health education element also reflected the Health Belief Model including perceived barriers, benefits, threat, self-efficacy and cues to action (40, 41). Typically, a Promotor greeted community members who drove up to the testing event, then asked whether the person was willing to hear a 5-min overview of COVID-19 preventive behaviors while waiting in the line of cars to get tested. If the community member was interested, the Promotor offered verbal instruction on effective mask wearing, hand washing, physical distancing, repeated testing, as well as the benefits of practicing these behaviors. After vaccines began to be widely available (approximately May 2021), the benefits of getting vaccinated was a fifth preventive behavior added to the health education element. During the verbal instruction, the Promotor presented a flier in the community member's preferred language with illustrations and brief explanations for each behavior (see Supplementary materials). Participants were invited to ask questions and share any concerns or hesitations. They were given a flier to take home and encouraged to bring a friend or family member to the next testing event.

The brief health education element closed with offering resources, and navigation support as needed, in recognition of the multifaceted barriers faced by Latinx community members in accessing services (2, 19). Materials for this resource navigation support included a customized list of local and state resources (e.g., food banks, social services, and information regarding funds for agricultural workers who missed work due to quarantine or positive test results) and informational handouts focused on how to prevent further viral spread if they or a family member tested positive.

Prior to launching the training of Promotores, a pilot test of the intervention was conducted at two testing events, with members of the research team serving as proxy Promotores. These experiences informed the scope and content of the Promotor training, as well as the order of operations at testing events. For example, the research team began by offering the health education after community members were tested, but at that point most community members had accomplished what they came for and declined the additional education in order to be on their way. When the research team changed to delivering the health education prior to community members getting tested, (thereby taking advantage of the time they were waiting in line to be tested), receptivity to the health education improved substantially.

### Promotor training

Over the course of the project, partner CBOs hired a total of 21 Promotores who met qualifications such as proficiency in Spanish and English, interpersonal skills, knowledge of cultural norms and community resources, access to internet and email, and computer literacy. Upon being hired, Promotores were directed to the project website for instructions on how to complete their seven-step training (see Table 3), which totaled approximately 6 hours of (paid) time. The Promotor training reviewed effective communication practices consistent with principles of trauma-informed care including safety, trustworthiness, transparency, collaboration, empowerment and intersectionality (3). These principles guided the design of the *Promotores de Salud* intervention, the processes at the testing events, the research team's ongoing interactions with Promotores, and how Promotores were trained to interact with community members. For example, safety was prioritized, with protocols to keep Promotores and community members safe from COVID-19 as well as from negative community responses to Promotores or the presence of Immigration and Customs Enforcement at a testing event. Trustworthiness and transparency were enhanced by working with community partners embedded in Latinx communities, being explicit in outreach materials that testing events were for *all* Latinx community members regardless of immigration status, and by excluding any questions about immigration status to minimize risk in a potential breach of confidentiality to testing registration data (55). Potential risks to Promotores (e.g., possible SARS-CoV-2 exposure) and testing participants (e.g., potential risk of breach of confidentiality) were reviewed in advance in the individual's preferred language. To further foster trust and collaboration with partners across sectors, the research team regularly acknowledged the limitations of their resources and knowledge base and expressed appreciation, warmth, and concern for collaborators, many of whom were spread very thin across professional and personal domains as they managed the pandemic. Each Promotor meeting highlighted the hard work, positive attitude, and/or creative problem-solving of Promotores, their CBOs, or the project as a whole.

**Table 3:** Promotor training overview.

**Step**	**Description**
1: Complete module	• Complete an online, free, and publicly available Community Health Worker COVID-19 Module provided in Spanish and English by the National Network of Public Health Institutes (53).
	• Module contents:
	° An introduction to COVID-19
	° How it is transmitted
	° Common symptoms
	° Preventive strategies
	° How to care for self or others who are sick
2, 3: Watch two asynchronous presentations	• Presentation origins: created and recorded by the research team in consultation with the Community and Scientific Advisory Board and informed by best-practice guidelines in how to train Promotores in the context of a research study (54).
	• Presentation contents:
	° Purpose and structure of the project
	° Research and safety protocols
	° Myths and facts related to COVID-19
	° The on-site testing process
	° How results would be shared
	° Recommended actions (a) while waiting for test results and (b) if someone tests positive for SARS-CoV-2
	° Roles and responsibilities of Promotores
	° Paperwork instructions related to tracking outreach efforts, intervention fidelity, and the number of community members to whom the health education was delivered
	° How to utilize principles of trauma-informed care and motivational interviewing in outreach and health intervention interactions
	° Brief knowledge assessment embedded in each presentation to solidify learning
4: Complete knowledge assessment	• Complete an online survey testing understanding of the content covered in the asynchronous presentations.
	• Score of 85% or higher required to proceed to next step.
	• Multiple retakes permitted until passing score is achieved.
5: Complete Individual Investigator Agreement	• Review, sign, and submit an Individual Investigator Agreement. • The agreement specifies Promotor roles and responsibilities for protection of human subjects, as members of the research team who deliver the intervention.
6: Visit a testing event	• Recommendation and encouragement to visit one of the project's testing events and to get tested, to obtain first-hand experience of the process prior to serving as a Promotor.
7: Review Resources	• Review project webpage resources and materials for Promotores including:
	° Example outreach and brief health education scripts
	° Health education flyers
	° Video demonstration in Spanish of a Promotor delivering the health education intervention element to a community member

The principle of collaboration was enacted through regular meetings with the CSAB, public health and community services team, and Promotores, ensuring on-going feedback loops that further refined the intervention. Intervention modifications suggested during these meetings were carefully considered, and a rationale provided for any suggestions not incorporated (e.g., such a change would be contrary to the study design specifications of the grant) or delayed (e.g., required modification to the IRB plan). Empowerment was supported by prioritizing the value of lived experience and community knowledge in the hiring of Promotores, and by trusting Promotores to select the outreach strategies and talking points that they deemed most effective for their communities. Promotores were also trained to honor community members' choices during outreach and brief health education interactions. Lastly, the principle of intersectionality was engaged by consistently acknowledging the structural and systemic constraints affecting Latinx communities and their experience of the pandemic, attending to heterogeneity of Oregon Latinx communities, and by respecting the importance of variation in how the intervention was deployed across communities regarding outreach strategies, community partners, talking points, languages spoken and written materials, and resources shared.

Promotors were trained in how to use motivational interviewing (MI) (56) in the context of their outreach and when delivering the brief health education. MI has demonstrated strong positive effects as a behavior change intervention among Latinx populations and in the context of health promotion interventions (57–59). Training addressed the relational elements, or the “spirit,” of MI as well as how Promotores could apply MI principles and core skills in their role (60). Training in the relational elements of MI emphasized the importance of approaching interactions with a compassionate, non-judgmental, and collaborative stance. For example, in response to ambivalence or resistance to getting tested, Promotores were trained to evoke change by exploring the person's ambivalence to get tested through open-ended follow-up questions, such as eliciting pros and cons of getting tested. Similarly, when delivering health education, Promotores were trained to refrain from pushing back on resistance to mask-wearing, and instead to explore this perspective and ask for permission to share information with the participant about the value of mask-wearing. Promotores engaged in these interactions using core MI skills, such as open-ended questions, affirmations regarding the person's current COVID-19 mitigating behaviors and their self-efficacy for change, reflective listening, and summarizing to convey empathy and compassion. Promotores training highlighted the importance of responding in accord with participants' readiness for testing, mask wearing, and other key COVID-19 preventive behaviors (61).

## Discussion

Guided by Duncan et al.'s (27) and Smith, Levkoff and Ory's (28) checklists, this community case study of the *Promotores de Salud* intervention development and delivery is shared here for the purposes of transparency, replicability, and furthering public health impact by facilitating intervention uptake among Latinx communities. Project strengths, lessons learned for future applications, and additional recommendations are highlighted below.

### Strengths

The intervention development process exemplified effective research-community partnerships. Namely, multi-disciplinary researchers, county and state public health practitioners, and leaders from Latinx-serving CBOs pooled their time, resources, connections, and methodological and substantive expertise to accomplish the shared goal of developing a culturally and trauma-informed intervention to promote SARS-CoV-2 testing and preventive behaviors among Oregon's Latinx communities. This project demonstrates how the spirit of CBPR can be infused throughout a public health intervention in spite of the challenges posed by time pressure and constraints of the funding mechanism. The continuous influence of our community social service and public health partners, advisory board, and Promotores greatly enhanced the quality of cultural tailoring, supported learning, and promoted shared ownership of the intervention's success (13).

Another strength was infusing trauma-informed care principles into this project beyond the development of the intervention and training of the Promotores. The pandemic itself was a source of trauma, generating a sense of fear and uncertainty across all communities, causing death and illness, interrupting and constraining daily activities, and restricting access to valuable social supports (62, 63). The strain of these conditions was amplified in Latinx communities but was, nonetheless, palpable across the entire project team. Such a context made it all the more important to curate a team of people who are kind, gracious, and non-defensive. Further, given the long history of research-community partnerships in which value is extracted from the community by researcher-experts, it was paramount that Latinx community members and leaders from Latinx-serving CBOs were upheld as the experts on their communities and their guidance was sought and trusted. Trusting builds trust. The research team acted more as facilitators of the work—synthesizing extant and newly collected data, drafting intervention elements and materials, arranging for on-going solicitation of feedback and direction from these community experts, and incorporating their feedback. Transparency and support for the autonomy of collaborators built safety. Inviting critique and responding with openness and humility increased the likelihood and quality of subsequent feedback. A research team member took diligent notes at all meetings with community partners. These notes, shared after each meeting, documented feedback, recommendations, and adaptations and tracked communication and responsivity over time, which also helped coordinate and communicate these efforts across a large project team. Prior to ending a meeting, a research team member took responsibility for following up on any questions or concerns that could not be addressed during the meeting. The project was a true partnership with a shared sense of urgency and mission.

Responsiveness to Promotores was prioritized, highlighting the critical value of their community knowledge and first-hand experiences delivering the intervention in the community. For example, within and outside of meetings, suggestions and ideas for enhancing outreach and education efforts were responded to promptly and the group was engaged in collaborative problem-solving of the challenges that arose on the ground. A warm relational climate was built in which Promotores and other partners felt comfortable sharing ideas and concerns. Gratitude and respect for their contributions to the project were consistently expressed.

Another strength was the robust representation of Latinx, bicultural, and/or bilingual researchers, professionals, and community members on the research team and CSAB. Leadership with intimate cultural knowledge, relevant lived experience, and Spanish-English language skills was instrumental in fostering trusting partnerships and effectively developing the *Promotores de Salud* intervention in a relatively short period of time. Meetings with some county CBOs and Promotores were conducted in Spanish, and in response to suggestions from Promotores, meeting minutes were disseminated in both English and Spanish.

Finally, sustainability was an important goal of the project. The partnerships and new lines of communication we established across entities and counties can facilitate greater responsivity to future community health concerns and endeavors in Oregon Latinx communities. The CSAB has expressed enthusiasm for continued service focused on other Latinx health projects. The *Promotores de Salud* intervention has utility beyond the work of this project. This intervention could accompany testing or vaccination events delivered by public health or other entities. Currently, for example, the intervention is being used at vaccination and testing events hosted by Oregon's Mexican Consulate. The elements of the *Promotores de Salud* intervention are also well poised for adapting to other community-wide disease prevention and control efforts focused on, for example, influenza or chronic diseases such as heart disease or type 2 diabetes.

### Lessons learned for future applications

Although the research-community partnership was a strength, it also presented challenges, often deriving from the rigorous and sometimes lengthy protocols that accompany federally grant-funded and human subjects research. For example, frustration sometimes arose when logical and valuable suggestions from partners could not be incorporated because of grant-related limitations (e.g., funds could not support vaccination efforts), requirements associated with human subjects research (e.g., approval of intervention changes), or research design (e.g., Promotores could not be deployed to all testing sites until completion of the data collection period for evaluating the wait-list control trial). We learned the importance of frequent meetings with CSAB, CBOs, and Promotores that allowed researchers opportunities to respond to frustration and provide the rationale and constraints of the research process. These regular channels of communication supported transparency and helped to increase understanding and patience among the groups.

The fast-paced nature of the project in the context of a pandemic also made it impossible for some of the partner CBOs to take on tasks or processes to the extent initially envisioned, even though financial resources were available. The partner CBOs were actively involved in the delivery of an array of services to their communities (e.g., proving economic resources, health and legal services, childcare, employment support). The increasing demand for these services and staffing shortages as a result of the pandemic stretched the already limited human resources of CBOs. This posed some challenges to supporting the readiness and capacity of CBOs, as well as the Promotores who worked for these CBOs, to deliver the intervention independent of the research team's involvement (13). As such, research team members took on additional, unanticipated aspects of the project in order to continue moving forward. In this context, the intervention development was well suited for a research-community partnership in which the university had substantial human resources to contribute.

We learned to adjust timelines for adaptations to intervention materials and/or Promotores training. Prompted by changing CDC guidelines or state ordinances regarding COVID-19 preventive behaviors, adaptations required drafting, obtaining feedback on, and translating new health education and training content, obtaining IRB approval, and then training CBOs and Promotores on new health education material. Moreover, even after training, Promotores' adoption of new health education content took time and often required review and re-review at the twice-monthly Promotores meetings. Similarly, when vaccines became available and testing rates declined, adaptation of the materials to emphasize the continued importance of SARS-CoV-2 testing took time.

Because of social distancing requirements and the physical dispersion of the project across nine counties, it was necessary to rely almost entirely on technology to communicate with each other and community partners. This was a particular challenge among those Promotores who were less comfortable engaging with the project's website. Ultimately, we found Zoom and email to be the best means of communication and resource sharing. Within the University research team, Zoom and Microsoft Teams were most used. We learned, although remote formats can make forming relationships challenging, it also offered the possibility to meet safely and regularly with people across the state.

### Additional recommendations

In order to more fully prioritize the suggestions and timelines of the community partners, we recommend that funding agencies interested in the use of CBPR build in flexibility into study aims to better reflect the priorities of community collaborators, especially in times of public health emergency when community contexts and needs can quickly change. We also recommend that research teams work with their IRB in the grant proposal stage to arrange a fast-track review of any study protocol adjustments for the duration of the study to minimize implementation delays. Despite the accelerated pace of RADx-UP funding timeline (4 months from request for proposals to notification of award), it was about a year between Oregon's shelter-in-place order (March 2020) and the implementation of *Promotores de Salud* in the community (February 2021). Funding agencies could consider an emergency mechanism for dispersing awards immediately to CBOs that serve vulnerable communities toward hiring additional personnel (even if remote employees) and toward research-community collaborations to facilitate faster acting CBPR-led public health responses.

The heterogeneity of Latinx communities means that local adaptations should be on-going, and we recommend close attention to language and literacy. We recommend not only bicultural and bilingual staff but Spanish native speakers' involvement in the creation and/or review of materials to produce accurately written messages that are culturally and linguistically responsive. We were unable to recruit a Mam-speaking Promotor and arranged for translators at testing sites where Mam-speaking community members were expected. Our written materials were not translated into Mam due to low Mam literacy rates in the community. Further, our materials adhered to a 6^th^-grade reading level for English and Spanish and incorporated visual cues, but still relied on textual information for certain aspects of outreach, health education, and resource navigation support. An agency specializing in multi-language audio and visual outreach services was contracted to create videos in Spanish and Mam. However, these products did not come to fruition due to agency staffing limitations. These conditions made our verbal outreach efforts (radio announcements and in-person presence at community locations) critical.

The *Promotores de Salud* intervention will also need ongoing adaptation as SARS-CoV-2 and its variants continue to change and scientific understanding of COVID-19 prevention and control evolves. We recommend generating the critical feedback for necessary adaptations *via* partnerships with public health practitioners, Latinx-serving CBOs, and Promotores in the communities where the intervention will be implemented. Evidence that this intervention resulted in significantly higher participation of Latinx community members in testing events (43) is very promising, warranting replication and continued evaluation.

## Conclusion

In response to disproportionate COVID-19 burden experienced among Latinx communities in Oregon, we collaboratively developed a trauma-informed and culturally tailored *Promotores de Salud* intervention to increase SARS-CoV-2 testing and preventive behaviors among Oregon's Latinx communities. This case study presents details of the development and refinement processes, the intervention itself, and the strengths and lessons learned of such public health field work to benefit future research-community partnerships and to facilitate replication of the *Promotores de Salud* intervention toward eliminating COVID-19 disparities among Latinx communities nationwide.

## Data availability statement

The de-identified data supporting the conclusions of this article will be made available by the authors, without undue reservation.

## Ethics statement

The studies involving human participants were reviewed and approved by University of Oregon Institutional Review Board. Written informed consent for participation was not required for this study in accordance with the national legislation and the institutional requirements.

## Author contributions

All authors made substantial contributions to the conception or design of the work. EB, CC, KY, AJ, and HT contributed substantially to the data acquisition, analysis, and interpretation. EM, SD, AM, MM, AN, KV, JG, OSJP CSAB, WC, DD, and LL contributed substantially to the data interpretation. EB, EM, SD, AM, KY, AJ, and HT drafted the work and CC, OSJP CSAB, and LL substantively revised it. All authors read and approved the final manuscript and agree to be accountable for the content of the work.

## Funding

Research reported in this publication was supported by the National Institute on Drug Abuse of the National Institutes of Health under Award Number P50 DA048756-02S2 (MPIs: LL, WC, and DD). The content is solely the responsibility of the authors and does not necessarily represent the official views of the National Institutes of Health. The funding body had no role in the design of the study and collection, analysis, and interpretation of data or in writing the manuscript.

## Conflict of interest

The authors declare that the research was conducted in the absence of any commercial or financial relationships that could be construed as a potential conflict of interest.

## Publisher's note

All claims expressed in this article are solely those of the authors and do not necessarily represent those of their affiliated organizations, or those of the publisher, the editors and the reviewers. Any product that may be evaluated in this article, or claim that may be made by its manufacturer, is not guaranteed or endorsed by the publisher.

## Supplementary material

Data Sheet 1 Example outreach card.

Data Sheet 2 Health education flier in Spanish.

Data Sheet 3 Health education flier in English.
