# Supplementary material for: Development and design of a culturally tailored intervention to address COVID-19 disparities among Oregon's Latinx communities: A community case study
Source: Front Public Health. 2022 Sep 23;10:962862. doi: 10.3389/fpubh.2022.962862 (PMC9541743; doi:10.3389/fpubh.2022.962862)
Supplement: Data Sheet 1 — Example outreach card. [file Data_Sheet_1.PDF]

OREGON SALUDABLE

Juntos Podemos

Hágase la prueba. Protéja su familia y la mía.
No se necesita seguro de salud y no se pide identificación.
En colaboración con [Nombre de la organización].

Get tested! Protect your family and mine.
No insurance or identification is needed.
In partnership with [Organization name].

Organization
Logo Here

Para obtener información sobre las ubicaciones y los horarios de los eventos de pruebas de COVID-19, visite: https://blogs.uoregon.edu/osjp/registro/

For COVID-19 testing event locations and times, visit: https://blogs.uoregon.edu/osjp/registro/

Puede registrarse aquí pero no es necesario hacerlo antes del evento. Use la cámara de su teléfono para obtener la página de registro.

You can register here but advance registration is not required. Use the camera on your phone to bring up webpage.
