# Supplementary material for: Development and design of a culturally tailored intervention to address COVID-19 disparities among Oregon's Latinx communities: A community case study
Source: Front Public Health. 2022 Sep 23;10:962862. doi: 10.3389/fpubh.2022.962862 (PMC9541743; doi:10.3389/fpubh.2022.962862)
Supplement: Data Sheet 2 — Health education flier in Spanish. [file Data_Sheet_2.PDF]

INFORMACIÓN SOBRE COVID-19

CUÍDATE Y CUIDA A LOS DEMÁS

Para seguir los guias estatales de salud pública visite: https://govstatus.egov.com/or-oha-cubrebocas

5 MEDIDAS PARA EVITAR EL CONTAGIO Y TRANSMISION DE COVID-19

1. PONGASE LA VACUNA DE COVID-19

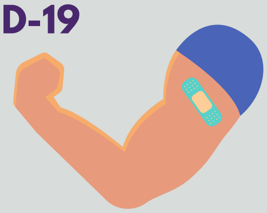

- Las vacunas son nuestra mejor defensa contra COVID-19 y su transmisión a nuestras familias y comunidad.
- Se a comprobado en miles de personas que la vacuna es segura. Recibiendo la vacuna no reemplaza otros comportamientos de salud presentados aquí.
- Siga las pautas de vacunación más recientes en el sitio web mencionado anteriormente.

2. HÁGASE LA PRUEBA DE COVID-19

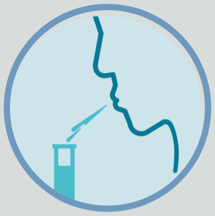

- Hacerse la prueba todavía es una manera muy importante de prevenir que se propague la enfermedad, independientemente del estado de vacunación. Por ejemplo, hágase la prueba si:
  - Tiene síntomas de COVID-19 (fiebre, tos),
  - Si ha estado en contacto cercano con alguien que no esta vacunado o tiene COVID-19,
  - Vive con niño/as que todavía no califican para vacunarse
  - Va a viajar a un lugar riesgoso
- Hacerse la prueba frecuentemente es importante si tiene enfermedades crónicas o si es persona de edad avanzada

3. CÚBRASE LA BOCA Y LA NARIZ CON UNA MASCARILLA

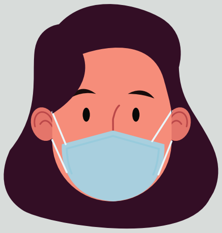

- Usando una máscara lo protege a usted y a los demás. Para seguir la guía más reciente sobre el uso de máscaras en espacios interiores y exteriores, visite el sitio web de OHA (enlace mencionado anteriormente).

4. LAVARSE LAS MANOS CON FRECUENCIA

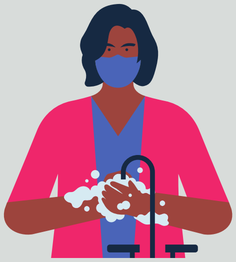

- Lávese las manos con frecuencia con agua y jabón por al menos 20 segundos, especialmente después de haber estado en un lugar público, o después de sonarse la nariz, toser o estornudar.

5. EVITE EL CONTACTO FÍSICO Y MANTENGA LA DISTANCIA

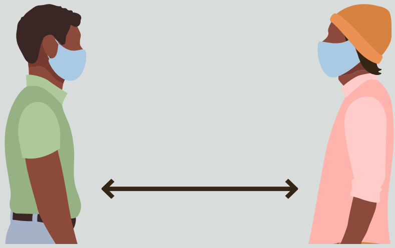

- Minimice el contacto cercano con otras personas que no viven en su hogar, especialmente cuando se encuentra en lugares interiores.
- Siga la guía más reciente sobre el distanciamiento físico visitando el sitio web de OHA (enlace mencionado anteriormente).

DESPUÉS DE LA PRUEBA DE COVID-19

¿CÓMO RECIBIRÉ MIS RESULTADOS?

- Si la prueba detecta el virus, le llamara la oficina de salud pública del condado en aproximadamente 4 dias. Esté pendiente de esta llamada.
- También recibirá los resultados de la prueba por correo, mensaje de texto o correo electrónico (dependiendo del método que seleccionó) dentro de 4 días hábiles.

EL VIRUS NO FUÉ DETECTADO EN LA PRUEBA, PERO ME SIENTO ENFORMO(A) Y/O ESTUVE EXPUESTO(A) AL VIRUS

- Continúe evitando contacto cercano con otras personas si se siente enfermo o cree que ha estado expuesto. Si no se siente bien, es posible que no se detecte el virus porque no ha pasado suficiente tiempo entre el momento que posiblemente estuvo expuesto y el momento que se hizo la prueba.
- Si ha estado expuesto a alguien que tuvo resultado positivos de la prueba:
  - *Si **no** está completamente vacunado*, póngase en cuarentena durante 5 días después de que estuvo expuesto a la persona infectada
  - *Aun si está completamente vacunado*, use una máscara y hágase la prueba el día 5 después de que estuvo expuesto a la persona infectada

MI PRUEBA ES POSITIVA. ¿AHORA QUÉ HAGO?

- Debe aislarse durante al menos 5 días, incluso si no tiene síntomas. Si tiene síntomas y continúan después de 5 días, aísle hasta que no tenga síntomas. Continúe usando una máscara durante 5 días adicionales después del aislamiento.
- Legalmente, no puede perder su trabajo por dar positivo. Considere comunicarse con el Centro de ayuda legal de Oregon: https://oregonlawhelp.org; (541) 485-1017
- Aunque la moratoria de desalojo ha terminado, todavía existen algunas protecciones en caso de que pierda todo o parte de sus ingresos debido a la pandemia. Visite www.OregonRentersRights.org para obtener información sobre sus derechos y protecciones como inquilino.

¿QUÉ ES AISLAMIENTO?

- Aislamiento significa mantenerse alejado de otras personas después de tener una infección confirmada. Si es posible, aíslese en un lugar en su hogar donde no tenga contacto con otra personas y pueda usar un baño separado, si posible. Cuando este en aislamiento, no comparta utensilios, y si no puede mantener distancia, use una máscara en todo momento.

¿QUÉ ES CUARENTENA?

- La cuarentena es permanecer en casa, lejos de las personas que no viven en su hogar, para evitar la propagación del virus a otras personas de su comunidad. Puede ponserse en cuarentena haciendo:
  - Evitar lo mas posible todo contacto con personas que viven fuera de su hogar,
  - No ir de compras, &
  - Tratar de ordenar comida a tu puerta, o tal vez algún vecino o familiar puede traerle las compras.

ESTAS PAUTAS DE AISLAMIENTO Y CUARENTENA SON CONSISTENTES CON LAS RECOMENDACIONES DEL CDC EN 1/3/2022. LLAME AL 211 PARA OBTENER INFORMACIÓN ACTUALIZADA.

OREGON SALUDABLE
Juntos Podemos

Para más información visite: https://blogs.uoregon.edu/osjp

January 2022
