# Supplementary material for: Development and design of a culturally tailored intervention to address COVID-19 disparities among Oregon's Latinx communities: A community case study
Source: Front Public Health. 2022 Sep 23;10:962862. doi: 10.3389/fpubh.2022.962862 (PMC9541743; doi:10.3389/fpubh.2022.962862)
Supplement: Data Sheet 3 — Health education flier in English. [file Data_Sheet_3.PDF]

INFORMATION ABOUT COVID-19

PROTECT YOURSELF AND OTHERS

To follow state public health guidelines visit: https://govstatus.egov.com/OR-OHA-COVID-19

5 WAYS TO STOP THE SPREAD OF COVID-19

1. GET THE COVID-19 VACCINE

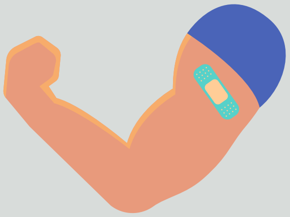

- Vaccines are our best defense against getting COVID-19 and spreading it to our families and community.
- The vaccine has been tested and proven safe. Receiving the vaccine does not replace other health behaviors presented here.
- Follow the latest vaccination guidelines on the website noted above.

2. GET TESTED FOR COVID-19

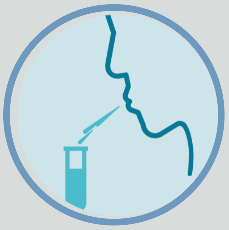

- Getting tested is still an important way to keep the illness from spreading, regardless of vaccination status. For example get tested if you:
  - have COVID-19 symptoms (fever, cough),
  - have come in close contact with someone who is unvaccinated or has COVID-19,
  - live with persons too young for vaccinations,
  - are traveling to a high-risk location
- Repeated testing is important if you have chronic health conditions or if you are an older adult.

3. COVER YOUR MOUTH & NOSE WITH A MASK

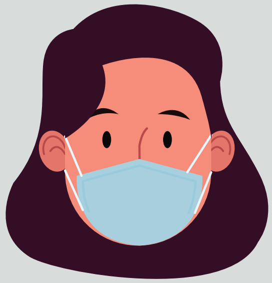

- Wearing a mask protects you and others. To follow the latest guidance on mask-wearing in indoor and outdoor spaces, visit the OHA website (link above).

4. WASH YOUR HANDS FREQUENTLY

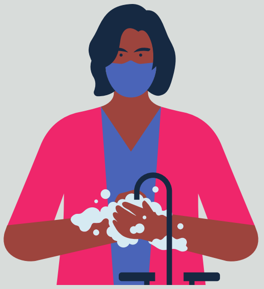

- Wash your hands often with soap and water for at least 20 seconds, especially after you have been in a public place, or after blowing your nose, coughing, or sneezing.

5. PHYSICAL DISTANCING

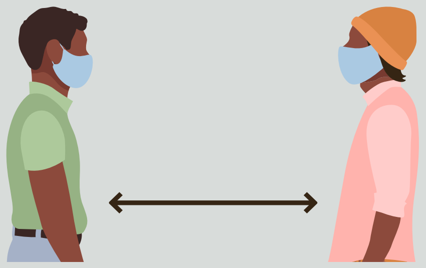

- Minimize close contact with others who do not live in your home, especially when you are indoors.
- Follow the latest guidance regarding physical distancing by visiting the OHA website (link above).

AFTER THE TEST FOR COVID-19

HOW WILL I RECEIVE MY RESULTS?

- If the test detects the virus, the county public health office may call you in about 4 days. Be on the look out for this call.
- You will also receive the test results by mail, text, or email (depending on the method you selected) within 4 business days.

THE VIRUS WAS NOT DETECTED IN MY TEST BUT I FEEL SICK OR WAS EXPOSED

- Continue to avoid close contact with others if you are feeling ill or think you have been exposed. The virus may not be detected because not enough time has passed between exposure and when you got tested.
- If you have been exposed to someone who tested positive:
  - *If **not** fully vaccinated, quarantine for 5 days after exposure and then get tested*
  - *Even if you are fully vaccinated, wear a mask and get tested day 5 after exposure*

MY TEST IS POSITIVE. WHAT DO I DO NOW?

- You should isolate yourself for at least 5 days even if you have no symptoms. If you have symptoms and they continue after 5 days, isolate until you no longer have symptoms. Continue to wear a mask for an additional 5 days after isolation.
- Legally, you cannot lose your job for testing positive. Consider contacting the Oregon Legal Aid Center: https://oregonlawhelp.org; (541) 485-1017.
- Even though the eviction moratorium has ended, there are still protections in place should you lose all or part of your income due to the pandemic. Visit www.OregonRentersRights.org for information on your rights and protections as a tenant.

WHAT IS ISOLATION?

- Isolation means staying away from others after a confirmed infection. If possible, isolate yourself in a place in your home where you will not have contact with other people and will be able to use a separate bathroom. When isolating, do not share utensils and, if you cannot maintain distance, wear a mask at all times.

WHAT IS QUARANTINE?

- Quarantine means stay home and away from people who do not live in your house to avoid spreading the virus. You can quarantine by:
  - Staying home,
  - Not going into any public places, like stores, restaurants, schools, or workplaces, &
  - Having a neighbor or family member bring you groceries or meals.

These isolation and quarantine guidelines are consistent with CDC recommendations on 1/3/2022. Call 211 for updated information.

OREGON SALUDABLE
Juntos Podemos

For more information visit: https://blogs.uoregon.edu/osjp

January 2022
